# Chronic intracranial hypertension after cerebral venous and sinus thrombosis – frequency and risk factors

**DOI:** 10.1186/s42466-021-00127-y

**Published:** 2021-05-17

**Authors:** Christina Geisbüsch, Christian Herweh, Christoph Gumbinger, Peter A. Ringleb, Markus A. Möhlenbruch, Simon Nagel

**Affiliations:** 1 Department of Neurology, Heidelberg University Hospital, Im Neuenheimer Feld 400, 69120 Heidelberg, Germany; 2 Department of Neuroradiology, Heidelberg University Hospital, Heidelberg, Germany

**Keywords:** Intracranial hypertension, Cerebral venous and sinus thrombosis, Visual impairment

## Abstract

**Background:**

Cerebral venous sinus thrombosis (CVST) can infrequently lead to chronical intracranial hypertension (IH) due to the altered venous drainage. The aim of this study was to ascertain the risk of IH after CVST and to stratify underlying risk factors.

**Methods:**

We performed a retrospective cohort analysis of all cases treated for acute CVST at our department between 2013 and 2019. IH was diagnosed at follow-up according to the modified Dandy criteria. CVST-patients with and without IH were descriptively compared conforming to available clinical and radiological data as well as outcomes.

**Results:**

Our study included 102 patients with acute CVST. In 70 cases complete follow-up data was available (68.6%). Seven of these patients developed symptomatic intracranial hypertension (10%; *N* = 7, *n* = 70) within a median follow-up of 6 months. Four of these patients (57.1% (*N* = 4, *n* = 7) vs. 3.2% (*N* = 2, *n* = 63); *p* < 0.001) presented recurrent sinus thrombosis in the further course. There were no significant differences between patients with or without IH concerning gender, age, risk factors, occluded vessels and treatment for their CVST. However the presence of visual deterioration at initial admission was higher in patients who developed IH afterwards (57.1% (*N* = 4, *n* = 7) vs. 20.6% (*N* = 13, *n* = 63); *p* = 0.03). Patients with chronic IH after CVST showed significantly less likely recanalization of the occluded vessel on follow-up MRI (no recanalization in 28.6% (*N* = 2, *n* = 7) vs. 4.8% (*N* = 3, *n* = 63); *p* = 0.02). All patients with IH had a good outcome (mRS 0–2) at discharge and follow-up.

**Conclusion:**

IH occurred in around 10% after CVST. Insufficient recanalization status may facilitate IH. Patients with visual disturbances seem to develop more likely IH afterwards. Patients who present IH after CVST may develop recurrent cerebral venous thrombosis.

## Introduction

Cerebral Venous Sinus Thrombosis (CVST) is a rare cause of stroke with an incidence from 1.3 to 1.6 per 100,000 persons [1] which often occurs in younger adults [2]. In comparison with other stroke subtypes, patients with CVST have generally a more favorable outcome; up to 80% are functional independent afterwards [3–5]. Clincal symptoms of CVST can range from headache to severe neurological complications such as loss of consciousness [2]. Moreover, patients may suffer from seizures, aphasia, visual disturbance, motor deficits and other clincial symptoms due to the localization of the thrombosis and its parenchymal lesions [2, 6]. Headache is the most frequent and in most cases the first symptom, whereby acute intracranial hypertension, venous congestion and infarction seem to play a key role [7]. Headache is also the key symptom of the more chronical course of idiopathic intracranial hypertension. Here, an increased cerebral pressure can be measured by lumbar puncture without evidence of structural reasons as stenosis of a sinus or postthrombotic constrictions [8]. Most prone to this condition are fertile and obese women who can present with visual deteroriations, papilledema and headache [8–10]. Diagnosis of idiopathic intracranial hypertension can be made according to Dandy criteria [9, 11]. The aetiology remains largely unknown [8], but lumbar punctures to decrease intracranial pressure, therapy with carbonic anhydrase inhibitors and weightloss are recommended to improve symptoms [9, 10]. CVST can also lead to chronical intracranial hypertension (IH) as a consequence of the altered venous drainage and can affect the overall outcome and quality of life. Previous studies already addressed the relationship between cerebral venous thrombosis and intracranial hypertension [12, 13]. The aim of our study was to estimate the risk of developing IH after CVST and to stratify underlying risk factors for this complication by analysis of a large cohort of CVST-patients with follow-up data from our institution.

## Methods

We performed a retrospective cohort study based on a systematical search in our electronical medical reports for patients who were treated for acute CVST at our Department of Neurology between January 1, 2013 and September 30, 2019. This study was approved by our local ethics committee (S-821/2019).

Patients with CVST were identified by the ICD-10 code (International Classification of Diseases, tenth revision) I67.6. CVST was diagnosed by magnetic resonance imaging (MRI) plus MR venography or computed tomography (CT) plus CT venography [14]. We recorded the following variables: demographic data, symptoms and signs from onset to diagnosis, CT- and MRI-findings, location of the thrombus, risk factors, treatment (acute and post-acute) and outcome at discharge and follow-up. The recanalization status was assessed by an unblinded neuroradiologist as primarily described by Stolz et al. [15]: no recanalization was assumed when the flow signal was still interrupted, a flow signal suggestive of a residual luminal narrowing of at least 50% was defined as partial recanalization and if the previously affected sinus had an uninterrupted flow signal with a residual luminal narrowing of < 50% it was evaluated as complete recanalization. Other imaging features were assessed as described in Herweh et al. [14].

Neurological outcome was assessed by an unblinded neurologist according to the modified Rankin Scale (mRS) categorized into independent (mRS 0–2) and dependent (mRS 3–6). All patients were admitted to the stroke unit or the neurointensive care unit and initially treated by either intravenous unfractioned heparin (with target partial thromboplastin time 50–80 s) or body-weight-adjusted low molecular weight heparin (LMWH). After the acute treatment an oral anticoagulation with vitamin K antagonist (VKA) phenprocoumon or with non-vitamin K antagonist oral anticoagulants (NOACs) was started. The specific anticoagulant was chosen individually by the treating physician or was based on the patient’s inclusion into trials (e.g. RESPECT-CVT [16]). The usual duration of the anticoagulation treatment was at least 6 months after the post-acute phase, but in selected cases and on individual basis the specific anticoagulant was changed during follow-up.

IH was diagnosed at follow-up according to modified Dandy criteria [9, 11], with exception of criterion 4 and in one patient criterion 5 due to relatively contraindicated lumbar puncture (see Table 1). For primary analysis of the recanalization status and clincal outcome at follow-up we selected the first follow-up visit in which a clinical examination and a MRI-scan took place (which was normally scheduled 6 months after CVST). If patients had more than one follow-up visit, we selected the first visit within the follow-up period in which both (a clinical examination and a MRI-scan) were performed for analysis of the recanalization status and clinical outcome. Other information such as recurrent CVST, treatment for IH and ophthalmological outcome was collected at every visit. Characteristics of CVST-patients with and without IH were then descriptively compared. Intracranial pressure, i.e. IH was measured by lumbar puncture and expressed as mm H^2^O (water).

**Table 1:** Modified Dandy criteria according to [9, 11]

1	Symptoms of raised intracranial pressure (headache, nausea, vomiting, transient visual obscurations, or papilledema)
2	No localizing signs with the exception of abducens (sixth) nerve palsy
3	The patient is awake and alert
4	Normal CT/MRI findings without evidence of (acute) thrombosis
5	LP opening pressure of > 25 cmH2O and normal biochemical and cytological composition of CSF
6	No other explanation for the raised intracranial pressure

### Statistical analysis

We considered demographic, clinical, imaging and treatment variable as possible explanatory variables of developing chronical IH after CVST. Continuous and ordinal variables were calculated as median with range (min-max). Bivariate analyses were performed for each categorical variable with the Chi-Square-test and Mann-Whitney-U-Test for continuous variables after testing for normal distribution (with Shapiro-Wilk Test). The level of significance was set at 0.05 (two-sided) and was calculated for all statistical tests. Since no adjustment for multiplicity was performed, the *p*-values need to be interpreted only descriptively and do not allow confirmatory statements. Analysis were conducted using SPSS 25.0 (Armonk, NY: IBM Corp.). The data that support the findings of this study are available from the corresponding author upon reasonable request.

## Results

One hundred and two patients with diagnosed acute CVST according to the aforementioned criteria were enrolled in this study (Table 2). Three patients already died during the acute-phase of the CVST, 8 patients had a clinical follow-up without imaging information and 21 more patients were lost to follow-up. We were able to obtain complete data (including follow-up MRI and clinical examination) of 70 patients with a median follow-up of 6 months (range, 2–42 months). Median age of all patients (*n* = 70) at time of admission was 40.5 years (range 17–77 years) and around 73% of all patients were female. Seven CVST-patients developed symptomatic intracranial hypertension (10%; *N* = 7, *n* = 70) according to the modified Dandy criteria which led to a presentation at the emergency department or at the outpatient clinic.

**Table 2:** Characteristics of all CVST-patients, all CVST-patients with Follow-up Data, CVST- patients without IH and with IH. Age is expressed as median (min-max) with *Mann-Whitney-U-Test, categorical data expressed as n (%) with Chi-Square-Test

	All CVST (***n*** = 102)	All CVST with Follow-up Data (***n*** = 70)	CVST without IH (***n*** = 63)	CVST with IH (***n*** = 7)	***p***-value
Gender female	75 (73.5%)	51 (72.9%)	46 (73%)	5 (71.4%)	0.93
Age, years	40.5 (17–77)	40.5 (17–77)	41 (17–77)	40 (19–67)	0.83*
**Symptoms and signs**					
Headache	84 (82.4%)	58 (82.9%)	52 (82.5%)	6 (85.7%)	0.83
Isolated headache	23 (22.5%)	19 (27.1%)	17 (27%)	2 (28.6%)	0.93
Visual disturbance	20 (19.6%)	17 (24.3%)	13 (20.6%)	4 (57.1%)	0.03
Paresis	25 (24.5%)	16 (22.9%)	15 (23.8%)	1 (14.3%)	0.57
Aphasia	16 (15.7%)	7 (10%)	7 (11.1%)	–	0.35
Focal seizures	14 (13.7%)	12 (17.1%)	11 (17.5%)	1 (14.3%)	0.80
Seizure with generalization	23 (22.6%)	12 (17.1%)	12 (19%)	–	0.20
Any seizure	37 (36.3%)	26 (37.1%)	25 (39.7%)	1 (14.3%)	0.19
Mental status disorder	14 (13.7%)	8 (11.4%)	7 (11.1%)	1 (14.3%)	0.80
Disturbed consciousness	13 (12.7%)	4 (5.7%)	4 (6.3%)	–	0.49
**CT/MR findings**					
Infarct one hemisphere	19 (18.6%)	11 (15.7%)	11 (17.5%)	–	0.23
Infarct both hemispheres	5 (4.9%)	2 (2.9%)	2 (3.2%)	–	0.63
Hemorrhage one hemisphere	35 (34.3%)	21 (30%)	18 (28.6%)	3 (42.9%)	0.43
Hemorrhage both hemispheres	9 (8.8%)	4 (5.7%)	4 (6.3%)	–	0.49
**Occluded sinus/vein**					
Superior sagittal sinus	43 (42.5%)	30 (42.9%)	28 (44.4%)	2 (28.6%)	0.42
One/Both lateral sinus	75 (73.5%)/7 (6.9%)	53 (75.7%)/5 (7.1%)	47 (74.6%)/4 (6.3%)	6 (85.7%)/1 (14.3%)	0.52 / 0.44
Straight sinus	14 (13.7%)	9 (12.9%)	8 (12.7%)	1 (14.3%)	0.91
Inner cerebral veins	8 (7.8%)	3 (4.3%)	3 (4.8%)	–	0.56
Cortical veins	23 (22.6%)	17 (24.3%)	16 (25.3%)	1 (14.3%)	0.52
Jugular veins	36 (35.3%)	28 (40%)	24 (38.1%)	4 (57.2%)	0.33
Multiple thrombosis in more than one dural sinus/vein	62 (60.8%)	48 (68.6%)	43 (68.3%)	5 (71.4%)	0.86
Thrombus extending from lateral sinus into jugular vein	34 (33.3%)	27 (38.6%)	23 (36.5%)	4 (57.2%)	0.29
**Risk factors**					
Thrombophilia	28 (27.5%)	20 (28.6%)	19 (30.2%)	1 (14.3%)	0.38
Malignancy	11 (10.8%)	5 (7.1%)	4 (6.3%)	1 (14.3%)	0.44
Pregnancy	1 (1%)	1 (1.4%)	1 (1.6%)	–	0.74
Puerperium	1 (1%)	–	–	–	–
Oral contraceptives	25 (24.5%)	21 (30%)	19 (30.2%)	2 (28.6%)	0.93
Vaginal ring	6 (5.9%)	6 (8.6%)	6 (9.5%)	–	0.39
Steroid therapy	13 (12.7%)	9 (12.9%)	9 (14.3%)	–	0.28
Cytotoxic therapy	8 (7.8%)	4 (5.7%)	4 (6.3%)	–	0.49
Smoking	9 (8.8%)	7 (10%)	6 (9.5%)	1 (14.3%)	0.69
Head injury	4 (3.9%)	3 (4.3%)	3 (4.8%)	–	0.56
**Treatment – acute phase**					
LMWH	74 (72.5%)	55 (78.6%)	49 (77.8%)	6 (85.7%)	0.63
Heparine i.v.	24 (23.5%)	13 (18.6%)	13 (20.6%)	–	0.18
Hemicraniectomy	7 (6.9%)	4 (5.7%)	4 (6.3%)	–	0.49
Mechanical thrombectomy	11 (10.8%)	4 (5.7%)	3 (4.8%)	1 (14.3%)	0.30
**Treatment – post-acute phase**					
OAK	39 (38.2%)	29 (41.4%)	26 (41.3%)	3 (42.9%)	0.94
NOAK	44 (43.1%)	35 (50%)	31 (49.2%)	4 (57.1%)	0.69
Rivaroxaban	25 (24.5%)	21 (30%)	19 (30.2%)	2 (28.6%)	0.93
Dabigatran	18 (17.6%)	13 (18.6%)	12 (19%)	1 (14.3%)	0.76
**Outcome at discharge**					
mRS 0–2 at discharge	75 (73.5%)	58 (82.9%)	51 (81%)	7 (100%)	0.20
**Outcome at follow-up**					
mRS 0–2 at follow-up		65 (92.9%)	58 (92.1%)	7 (100%)	0.44
Complete recanalization		18 (25.7%)	16 (25.4%)	2 (28.6%)	0.86
Partial recanalization		47 (67.1%)	44 (69.8%)	3 (42.9%)	0.15
No recanalization		5 (7.1%)	3 (4.8%)	2 (28.6%)	0.02
Recurrent sinus thrombosis		6 (8.6%)	2 (3.2%)	4 (57.1%)	< 0.001

Three of these seven patients (42.9%; *N* = 3, *n* = 7) presented headache and visual deterioration as symptoms of IH at a median of 7 months after CVST (1–21 months, Table 3). In two of these three patients this combination of symptoms had simply persisted after their acute CVST. The other patient presented isolated headache at diagnosis of CVST and developed visual problems afterwards. Three patients (42.9%; *N* = 3, *n* = 7) only reported visual disturbances without headache at follow-up: One of these 3 patients never had any kind of headache, the second patient developed visual worsening several weeks after CVST and had isolated headache when CVST was diagnosed and the third patient presented headache and visual disturbances as CVST-symptoms. The seventh patient complained about remaining headaches at follow-up whereas CVST had caused various symptoms such as focal seizures, dysarthria and paresis without any headache or visual symptoms before.

**Table 3:** Characteristics of chronic IH for each patient

	Patient 1	Patient 2	Patient 3	Patient 4	Patient 5	Patient 6	Patient 7
Time of IH diagnosis	2 months after CVST	7 months after CVST	8 months after CVST	11 months after CVST	1 month after CVST	5 months after CVST	21 months after CVST
Symptoms of IH	headache double vision, papilledema	headache, visual disturbances	headache, visual disturbances	visual disturbances, papilledema	visual disturbances	visual disturbances	Headache, delirium
Symptoms of previous CVST	headache, visual disturbances	isolated headache	headache, visual disturbances	isolated headache	headache, visual disturbances	visual disturbances	headache, paresis, focal seizures
Recanalization status	partial	no recanalization	complete	partial	complete	no recanalization	partial
CVST recurrence	yes (9 months after CVST)	yes (1 month and 12 months after CVST)	no	yes (12 months after CVST)	no	no	yes (in the further course) plus Dural fistulas
CSF pressure – at the time of diagnosis	500 mmH^2^O	330 mmH^2^O	270 mmH^2^O	410 mmH^2^O	550 mmH^2^O	500 mmH^2^O	not performed
Treatment of IH	initially acetazolamide and frequently lumbar punctures; due to remaining symptoms after insufficient attempt of mechanical recanalization, shunt-operation	acetazolamide	acetazolamide	intolerant to acetazolamide, short-time therapy with torasemide	repeated lumbar punctures	initially acetazolamide, but due to increasing CSF pressure and papilledema shunt-operation	insufficient attempt of mechanical recanalization, embolization of fistulas
Outcome (mRS)	symptom-free (mRS 0)	improved symptoms but remaining headaches (mRS 1)	symptom-free (mRS 0)	symptom-free (mRS 0)	symptom-free (mRS 0)	improved symptoms (mRS 1)	improved symptoms (mRS 2)
Ophthalmological Control	improved papilledema	improved papilledema	no abnormalities	not performed	not performed	improved papilledema on the right, severe left papillary atrophy	not performed

Median cerebrospinal fluid opening pressure of these seven patients was 455mmH^2^O (270–550 mmH^2^O). Lumbar punctures of patients with CVST who did not develop signs of IH in the further course were only performed in two cases and intracranial pressure was not measured. Four of the patients who developed chronic IH (57.1% vs. 3.2%; *p* < 0.001) presented recurrent sinus thrombosis in the further course (Table 3). But there were no significant differences between patients with or without IH concerning gender, age, risk factors for developing a CVST and the specific occluded vessels. In addition there were no significant alterations in treatment for the CVST and changes of the anticoagulation regime due to MRI findings at follow-up between these groups. However, the presence of visual deterioration at initial admission for CVST was higher in patients who developed IH afterwards (57.1% (*N* = 4, *n* = 7) vs. 20.6% (*N* = 13, *n* = 63); *p* = 0.03). All patients with IH had a functional independent outcome (mRS 0–2) at initial hospital discharge and follow-up, whereas 81% of all patients without IH were functional independent at initial discharge and 92% were functionally independent at follow-up. Patients with IH showed significantly more often no recanalization of the occluded vessels on follow-up MRI (28.6% (*N* = 2, *n* = 7) vs. 4.8% (*N* = 3, *n* = 63); *p* = 0.02). Four of the five patients with no recanalization (two patients with IH and two patients without IH) still had an extensive thrombosis of the left sinus transversus and sigmoideus intruding the Vena jugularis at follow-up. Only one patient presented at follow up with continued occlusion of a bridging vein.

Treatment of IH in this cohort was heterogenic and individualized for each patient (Table 3). Five patients were treated with acetazolamide. For two of these, this therapy was sufficient and improved their symptoms, whereas three cases required additional treatment or intervention. In two patients who were initially treated with acetazolamide ventriculo-peritonital shunting was necessary due to refractory headaches, optic atrophy with potential visual loss. One of these five patients showed intolerance to acetazolamide which is why therapy with torasemid was started instead and the anticoagulation therapy with NOAC (dabigatran) was resumed. In one case therapeutic lumbar punctures were temporarily repeated and the anticoagulation therapy was changed from vitamin K antagonist to dabigatran. In another patient endovascular therapy was attempted promptly after recurrent CVST (partial recanalization after 1st CVST), but unfortunately neither mechanical thrombectomy nor stenting of an underlying stenosis was technically possible. This patient then developed cerebral AV-fistula in the further course and was successfully treated with recurrent embolisations. In five cases the symptoms related to IH were reduced, one patient remained with a severe papillary atrophy without any further visual worsening and one patient who is still under follow-up suffers from continuing headaches with improved papilledema and visual symptoms in partial remission.

## Discussion

In our study 10% of CVST-patients developed chronical IH according to the modified Dandy criteria in the further course and received specific treatment for this complication despite having a functional independent outcome at discharge and follow-up. We identified as underlying risk factors no recanalization on follow-up MRI and the presence of visual symptoms at the initial diagnosis of CVST. Patients who developed IH afterwards also seemed to be prone to recurrent sinus thrombosis. IH is a clinical complication after CVST that can influence the overall outcome and can have a relevant impact on the quality of life [17], even though affected patients often present a functional independent neurological outcome as measured with the mRS.

Acute CVST can mimick idiopathic IH. In a study of Biousse et al. 59 patients out of a cohort of 160 consecutive CVST-patients (37%) only presented symptoms of IH such as headache with or without visual deterioration as signs for their acute CVST; lumbar puncture with measurement of the opening pressure was performed in 32 of these 59 CVST-patients and a raised intracranial pressure was confirmed in 25 cases (78%) [18]. In a subgroup analysis of the International Study on Cerebral Vein and Dural Sinus Thrombosis (ISCVT) raised intracranial pressure was found in 83.3% of 127 patients [19]. Hence, IH in patients with acute CVST is very common and a major pathophysiological mechanism to explain disease related symptoms.

Yet, up to date, it remains uncertain how many patients develop symptomatic, chronic IH in the further course after CVST and which patients may be under increased risk to do so. In our study, 10% of patients presented with isolated symptoms of IH at a median time of 7 months (range 1–21 months) after their CVST. It is likely that in the majority of the remaining patients the initially raised intracranial pressure slowly returned to normal with recanalization of the occluded vessel and/or establishment of collateral venous flow. Interestingly, there were no significant differences between CVST-patients with or without IH concerning gender, age, risk factors for developing a CVST in the first place, specific occluded vessels or anticoagulation treatment for the CVST. Most of our patients had a complete or partial recanalization at follow-up and only five of all CVST-patients (7.1%) had no recanalization. These recanalization rates correspond with other studies [20, 21]. However, and supporting the above mentioned hypothesis, patients with failed recanalization despite anticoagulation had more often IH as a complication than others. Therefore a lack of recanalization seem to be a risk factor for IH after first CVST. While some studies could not find any association between functional neurological outcome and recanalization status [14, 15, 20–23], a systematic review and meta-analyasis of 19 studies could show that a lack of venous recanalization was associated with a worse clinical outcome [24]. Another retrospective observational multicenter cohort study with 508 patients also revealed an association between recanalization status and functional outcome [25]. Some authors suggest that residual headache, i.e. persistent IH, is more common in patients with no recanalization [22, 26]. One study showed that complete recanalization rates were lower in patients with multiple thrombosis in more than one dural sinus [20]. Whereas some authors could draw the conclusion that thrombosis of the superior sagittal sinus was a positive predictor of recanalization [14], in another study the sigmoid sinus recanalized more often [21]. Among the five patients in our study that did not show any recanalization at follow up, 80% had an extensive thrombosis from lateral sinus into jugular vein. A recent study could also show that there were no differences between patients that were treated with dagibatran or warfarin concerning recanalization rates [23].

Our findings also lead to the assumption that patients who present visual disturbances as a symptom of their CVST seem to develop symptomatic IH more likely afterwards. Visual symptoms are described as a common complication of CVST-associated IH [27, 28]. According to Ding et al. there is a clear association of the magnitude of intracranial pressure and visual loss; a pressure ≥ 330mmH^2^O may be a cut-off value that predicts visual damage in CVST-patients [27]. Although these values were derived from a cohort of acute CVST-patients, the high cerebrospinal fluid (CSF) opening pressure (median 455mmH^2^O, 270–550 mmH^2^O) that was measured in our CVST-patients with chronic IH at follow up indicates a clear association with the increased frequency of visual symptoms in this subset of patients. Hence, as a clinically relevant consequence, patients with CVST and visual symptoms at presentation or during follow-up, should be closely monitored and medical treatment or interventions for IH should be considered early in these cases to avoid further visual deteroriation.

The present data cannot help to further elucidate the differences between patients who suffer from idiopathic IH versus those who develop IH as a secondary event. Features of patients with IH originating from idiopathic cause or from CVST, including CSF opening pressure, are very similar and the two entities may only be differentiated by imaging of the intracranial venous system [12, 29, 30]. However, isolated raised intracranial pressure from CVST differs in management from IIH and should be classified neither as “idiopathic IH” nor as “pseudotumor cerebri” [18]. The treatment of IH in our cohort was heterogenic (Table 3) and also included a switch in the anticoagulation regime for CVST in two patients.

This study has some limitations. First, a possible source of bias is the retrospective design of this study. Second, due to the prevalence of CVST the cohort was relatively small (*n* = 102) and there was a lack of complete follow-up data for some CVST-patients (lost-to-follow-up, *n* = 32). The reason for the lack of follow-up data remains unclear. One possible explanation could be that patients applied for post-acute follow-up appointments with physicians closer to their home. Another interpretation of the missing data could be that patients with very severe CVST or no symptoms at all after CVST might miss follow-up visits more likely. For 8 patients we obtained clinical data without imaging information, five of these patients showed an independent outcome at follow-up, two patients were dependent and one patient died due to other reasons. Furthermore we were not able to receive information for 21 patients (neither neurological nor neuroradiological). Twelve of these 21 patients had an independet outcome at hospital discharge whereas 9 patients were functional dependent at that point of time. These numbers seem to underline the above mentioned hypothesis. Third, lumbar punctures were not systematically performed on all CVST-patients at follow-up, but only when IH was clinically suspected by the treating physician. In one IH-patient lumbar puncture was not performed due to secondary dural fistulas (Table 3). Third, the fact that our IH-patients presented with very high CSF opening pressure (median 455mmH^2^O) suggests that we might have missed less severe cases of IH. Therefore the prevalence of IH might be higher than reported.

## Conclusions

IH is not a rare complication in the further course after CVST with possibly serious consequences to patients’ visual function. Therefore a follow-up visit including thorough anamnesis, clinical examination and a MRI scan with venography should be performed for each CVST-patient. Since visual symptoms and failed recanalization seem to indicate an increased risk of IH after CVST, an ophthalmologic consultation and diagnostic lumbar punctures should be considered in these cases to identify patients who might require specific IH treatment next to their anticoagulation regime.

## Data Availability

The data that support the findings of this study are available from the corresponding author upon reasonable request.

## Abbreviations

CVST: Cerebral venous sinus thrombosis
IH: Intracranial hypertension
mRS: Modified Rankin Scale
LMWH: Low molecular weight heparin
VKA: Vitamin K antagonist
NOACs: Non-vitamin K antagonist oral anticoagulants
